# HBx facilitates ferroptosis in acute liver failure via EZH2 mediated SLC7A11 suppression

**DOI:** 10.1186/s12929-021-00762-2

**Published:** 2021-10-06

**Authors:** Guo-Zhen Liu, Xu-Wen Xu, Shu-Hui Tao, Ming-Jian Gao, Zhou-Hua Hou

**Affiliations:** 1 Department of Infectious Diseases, Xiangya Hospital, Central South University, No.87, Xiangya Road, Kaifu District, Changsha, 410008 Hunan China; 2 Department of Infectious Diseases, Nanfang Hospital, Southern Medical University, Guangzhou, 510515 Guangdong China; 3 Department of Liver Diseases, Shenzhen Hospital, Southern Medical University, Shenzhen, 518100 Guangdong China

**Keywords:** Acute liver failure, Ferroptosis, HBx, EZH2, SLC7A11

## Abstract

**Background:**

Acute liver failure (ALF) is a syndrome of severe hepatocyte injury with high rate of mortality. Hepatitis B virus (HBV) infection is the major cause of ALF worldwide, however, the underlying mechanism by which HBV infection leads to ALF has not been fully disclosed.

**Methods:**

D-GalN-induced hepatocyte injury model and LPS/D-GalN-induced ALF mice model were used to investigate the effects of HBV X protein (HBx) in vitro and in vivo, respectively. Cell viability and the levels of Glutathione (GSH), malondialdehyde (MDA) and iron were measured using commercial kits. The expression of ferroptosis-related molecules were detected by qRT-PCR and western blotting. Epigenetic modification and protein interaction were detected by chromatin immunoprecipitation (ChIP) assay and co-immunoprecipitation (co-IP), respectively. Mouse liver function was assessed by measuring aspartate aminotransferase (AST) and alanine aminotransferase (ALT). The histological changes in liver tissues were monitored by hematoxylin and eosin (H&E) staining, and SLC7A11 immunoreactivity was assessed by immunohistochemistry (IHC) analysis.

**Results:**

D-GalN triggered ferroptosis in primary hepatocytes. HBx potentiated D-GalN-induced hepatotoxicity and ferroptosis in vitro, and it suppressed SLC7A11 expression through H3K27me3 modification by EZH2. In addition, EZH2 inhibition or SLC7A11 overexpression attenuated the effects of HBx on D-GalN-induced ferroptosis in primary hepatocytes. The ferroptosis inhibitor ferrostatin-1 (Fer-1) protected against ALF and ferroptosis in vivo. By contrast, HBx exacerbates LPS/D-GalN-induced ALF and ferroptosis in HBx transgenic (HBx-Tg) mice.

**Conclusion:**

HBx facilitates ferroptosis in ALF via EZH2/H3K27me3-mediated SLC7A11 suppression.

## Background

Acute liver failure (ALF) is a syndrome of severe hepatocyte injury characterized with rapid-onset elevation of aminotransferases and disturbed coagulation and mentation [1]. Although the mortality of ALF declines with the widespread availability of liver transplantation, the clinical outcomes remain unfavorable due to the major limitations of liver transplantation, such as organ shortage, infection and organ rejection [1, 2]. In addition, hepatitis B virus (HBV) infection is one of the leading cause of ALF worldwide [1, 3], patients with HBV-associated ALF exhibited low transplant-free survival [1]. Therefore, it is urgent to unravel the underlying mechanism by which HBV infection leads to ALF.

Ferroptosis is characterized by iron-driven lipid peroxidation and accumulation of reactive oxygen species (ROS) [4]. Dysregulated ferroptosis is implicated in diverse pathological processes, including cancer, neurological disorders, acute renal failure and hepatic injury [4, 5]. In particular, emerging evidence suggests that ferroptosis is involved in various liver diseases, such as drug-induced liver injury, viral hepatitis and ALF [6–8]. For instance, HMGB1 inhibitor glycyrrhizin alleviates ferroptosis in ALF via Nrf2/HO-1/HMGB1 signaling [6]. Ferroptosis also contributes to acetaminophen-induced ALF [8]. In addition, D-GalN/LPS is a well-known model of ALF [9–11]. Oxidative stress has been recognized as a key contributor to D-GalN/LPS-induced hepatotoxicity [12]. Given the role of ferroptosis in oxidative stress, we speculated that D-GalN might trigger ferroptosis to induce accumulation of ROS, thus leading to ALF. The regulatory mechanism of ferroptosis in ALF merits further investigation.

HBV X protein (HBx), an essential HBV regulatory protein, plays important roles in the development of HBV-associated severe liver disease, including hepatocellular carcinoma (HCC), liver fibrosis and ALF [13–15]. It is worth noting that HBx is associated with oxidative stress and lipid peroxidation, thereby contributing to liver pathogenesis [16–18], raising the possibility that HBx might be involved in ALF pathogenesis via modulating ferroptosis. In addition, it has been reported that HBx-induced aberrations in DNA methylation contribute to HCC tumorigenesis by modulating host gene expression [19]. For instance, HBx upregulates UCA1 via recruiting enhancer of zeste homolog 2 (EZH2), thereby suppressing p27Kip1 through histone H3 lysine 27 tri-methylation (H3K27me3) on its promoter in HCC cells [20]. A recent study has demonstrated that histone demethylase KDM3B protects against ferroptosis via increasing the expression of solute carrier family 7 membrane 11 (SLC7A11) which is a key component of the cysteine/glutamine antiporter system Xc^−^ [21]. Decrease of SLC7A11 results in dysregulated cysteine uptake and glutathione (GSH) biosynthesis, further leading to glutathione peroxidase 4 (GPX4) suppression and ferroptosis activation [5]. Interestingly, our preliminary results revealed that HBx induced significant enrichments of EZH2 and H3K27me3 in SLC7A11 promoter. This finding thus promotes us to hypothesize that HBx might trigger ferroptosis via EZH2/H3K27me3-mediated SLC7A11 suppression.

In this study, we illustrated that D-GalN triggered ferroptosis in primary hepatocytes. HBx potentiated D-GalN-induced hepatotoxicity and ferroptosis in vitro, and it suppressed SLC7A11 expression through H3K27me3 by EZH2. In addition, EZH2 inhibition or SLC7A11 overexpression reversed the effects of HBx on D-GalN-induced ferroptosis. In LPS/D-GalN-induced ALF mice model, the ferroptosis inhibitor ferrostatin-1 (Fer-1) protected against ALF and ferroptosis, whereas HBx exerted opposite effects on ALF and ferroptosis in HBx transgenic (HBx-Tg) mice.

## Methods

### Isolation of primary mouse hepatocyte and establishment of D-GalN-induced hepatocyte injury model

Primary hepatocytes were isolated from C57BL/6 mice (male, 6–8 week-old, b.w. 18 ~ 23 g; SJA Laboratory Animal Co Ltd., Changsha, Hunan, China) and characterized as previously described [22]. All animal studies were approved by the Ethical Committee of Central South University. In brief, in situ liver perfusion was conducted using HBSS containing 25 mM HEPES and 0.5 mM EGTA (Gibco, Thermo Fisher Scientific, Grand island, NY, USA), followed by the digestion of liver using collagenase buffer. After perfusion, liver was collected, and released hepatic cells were then filtered through a cell strainer (100 μm) and centrifuged. The pellets were resuspended in DMEM supplemented with 10% FBS (Gibco) for primary mouse hepatocyte culture. Cultures were grown at 37 ℃ with 5% CO_2_. The purity of hepatocyte was validated by immunofluorescent staining of the hepatocyte marker albumin as described [22]. To establish the D-GalN-induced hepatocyte injury model, primary hepatocytes were incubated with 50 mM D-GalN for 0, 1, 3 and 6 h. The 6 h incubation period was selected for subsequent experiments.

### Plasmid construction, lentiviral transduction, transfection and treatment

The full-length of mouse HBx and SLC7A11 were cloned into pcDNA3.1 vector (GenePharma, Shanghai, China). Vector alone served as a negative control. Si-NC and si-EZH2 were purchased from GenePharma. Lentiviral transduction or siRNA transfection was conducted using Lipofectamine 3000 transfection reagent (Invitrogen). Cells were treated with erastin (10 μM, Sigma-Aldrich, St Louis, MO, USA), Fer-1 (1 μM, Sigma-Aldrich), GSK126 (5 μM, Selleckchem, Houston, TX, USA) or CHX (50 μg/ml, Sigma-Aldrich).

### Cell viability assay

Cell viability was determined by MTT assay (Sigma-Aldrich). Briefly, primary mouse hepatocytes (5 × 10^3^) were plated into 96-well plates at 24 h prior to the designated treatment. At specified time points, MTT reagent (10 μl/well) was added into wells and incubated at 37 ℃. After 4 h, solubilization solution (100 μl/well) were then added into wells and the absorbance were measured at 570 nm wavelength using microplate reader (Bio-Rad, Hercules, CA, USA).

### Lipid peroxidation assay

Quantification of the oxidative stress marker malondialdehyde (MDA) was performed using Lipid Peroxidation (MDA) Assay Kit (Abcam, Cambridge, UK). In brief, TBA solution was incubated with standards and samples at 95℃ for 60 min. After being in ice bath for 10 min, the MDA-TBA adduct was analyzed at 532 nm wavelength using microplate reader (Bio-Rad).

### Glutathione (GSH) assay

The relative GSH level in cell or tissue extracts was determined using Glutathione Assay Kit (Sigma-Aldrich).

### Iron assay

The relative iron level in cell or tissue extracts was detected using an Iron Assay kit (Abcam, ab83366)according to the manufacturer’s instructions. In brief, firstly, cell or tissue extracts were added to an iron assay buffer, homogenized on ice, and then centrifuged at 13,000×*g* for 10 min at 4 ℃ to collect the supernatant. Secondly, a 50 μL sample was incubated with 50 μL of assay buffer in a 96-well microplate for 30 min at 25 ℃. The sample was then incubated with 200 μL of reagent mix in the dark for 30 min at 25 ℃, and the absorbance was measured at 593 nm with a microplate reader (Thermo Fisher Scientific).

### ROS detection assay

Liver tissue homogenate was prepared and subjected to ROS detection assay using Reactive Oxygen Species Detection Kit (Solarbio, Beijing, China) following the manufacturer’s protocol.

### ELISA assay

TNF-α, IL-6 and IL-1β in liver tissues were detected using commercial ELISA kits (Abcam) according to the manufacturer’s instructions.

### RNA isolation and qRT-PCR

Total RNAs were isolated from primary mouse hepatocyte using TRIzol reagent (Invitrogen). Reverse transcription was conducted using SuperScript IV Reverse Transcriptase (Invitrogen). qPCR reaction was performed using QuantiTect SYBR Green PCR Kit (Qiagen, Chatsworth, CA, USA). GAPDH served as an internal control for qRT-PCR. Data were calculated using 2^−ΔΔCT^ method. The primers were listed in Table 1.

**Table 1:** Primers list

Primer	Sequence 5′–3′
GPX4 sense	GTAACCAGTTCGGGAAGCAG
GPX4 anti-sense	TGTCGATGAGGAACTGTGGA
SLC11A2 sense	TGCGGAAGCTAGAAGCATTT
SLC11A2 anti-sense	CATGTTGTGTGGCATGATGA
SLC7A11 sense	TTGTTTTGCACCCTTTGACA
SLC7A11 anti-sense	AAAGCTGGGATGAACAGTGG
ACSL4 sense	CCGACCTAAGGGAGTGATGA
ACSL4 anti-sense	CCTGCAGCCATAGGTAAAGC

### Western blot

Protein extracts from primary hepatocytes were prepared in RIPA lysis buffer (Pierce, Thermo Fisher Scientific). Protein estimation was performed using BCA protein assay kit (Pierce). Proteins (30 μg) were separated by SDS-PAGE, and transferred onto PVDF membrane (Pierce). After blocking, the blots were incubated with primary antibody at 4℃ overnight, followed by the incubation with secondary antibody. The protein bands were visualized using SuperSignal Pico PLUS Substrate (Pierce), followed by detection with CCD detection system (Bio-Rad). The antibodies were listed in Table 2.

**Table 2:** Antibodies list

Antibody	Vendor	Catalog no.	Working dilution
GPX4	Abcam	ab125066	1:1000
SLC11A2	Abcam	ab55735	1:1000
SLC7A11	Abcam	ab37185	1:1000
ACSL4	Abcam	ab155282	1:10000
EZH2	Abcam	ab191250	1:1000
H3K27me3	Abcam	ab6002	1:1000
HBx	Abcam	ab39716,	1:1000
β-Actin	Abcam	ab8226	1:2000

Antibodies used in this study. Abcam, Cambridge, UK

### Chromatin immunoprecipitation (ChIP) assay

ChIP assay was performed using Pierce Agarose ChIP Kit (Pierce). In brief, transduced primary hepatocytes were cross-linked with 1% formaldehyde and lysed at 48 h post-transduction. Chromatin was digested using MNase, and followed by the incubation with anti-EZH2 (ab191250, Abcam), anti-H3K27me3 (ab6002, Abcam) antibody or normal IgG (negative control). DNA was purified and subjected to qRT-PCR analysis.

### Co-immunoprecipitation (co-IP)

Primary hepatocytes were lysed with Cell Lysis Buffer (Abcam). Pre-cleared protein extracts (1000 μg) were incubated with anti-EZH2 antibody (ab191250, Abcam) or normal IgG at 4℃ overnight, followed by the incubation with Protein A/G agarose (Santa Cruz Biotechnology, Santa Cruz, CA, USA) at 4℃ for 4 h. The protein complexes were then washed and subjected to western blot analysis. Whole cell lysates served as input control, and normal IgG acted as a negative control.

### Animal study

Male C57BL/6 mice (n = 20, 6–8-week-old, b.w. 18–23 g) were purchased from SJA Laboratory Animal Co Ltd., Changsha, Hunan, China. The ALF model was established as previously described [23]. In brief, mice were given 600 mg/kg D-GalN (Sigma-Aldrich) and 30 μg/kg LPS (Sigma-Aldrich) by intraperitoneal injection. Mice in Sham group were received saline injection. For Fer-1 treatment, mice were received 10 mg/kg Fer-1 at 1 h prior to D-GalN and LPS injection. The HBx transgenic (HBx-Tg) mice were generated as previously described [24]. The wild-type (WT) or HBx-Tg mice were then subjected to D-GalN and LPS administration. For GSK126 treatment, wild-type (WT) or HBx-Tg mice were received GSK126 (150 mg/kg) prior to D-GalN and LPS administration.

### Histopathological analysis

The liver tissues were harvested, fixed, embedded with paraffin and sectioned. For hematoxylin and eosin (H&E) staining, the sections were deparaffinized and stained with hematoxylin and eosin. For immunohistochemistry (IHC) analysis, sections were deparaffinized, rehydrated and subjected to antigen retrieval. After blocking, the slides were incubated with anti-SLC7A11 antibody (1:100; ab37185, Abcam) at 4℃ overnight. The slides were then incubated with HRP-conjugated secondary antibody, and visualized using AEC solution (Invitrogen).

### Assessment of liver function

Mouse liver function was assessed by measuring ALT and AST using Dimension RxL Max Integrated Chemistry System (Siemens Healthineers, Erlangen, Germany).

### Statistical analysis

Statistical analysis was conducted using the SPSS version 16.0 (SPSS, Chicago, IL, USA). All data were shown as the means ± standard deviation. Statistical analyses were performed using Student’s *t* test or one-way ANOVA. *P* < 0.05 was considered statistically significant.

## Results

### Ferroptosis is activated in D-GalN-induced hepatocyte injury model.

**Fig. 1:**
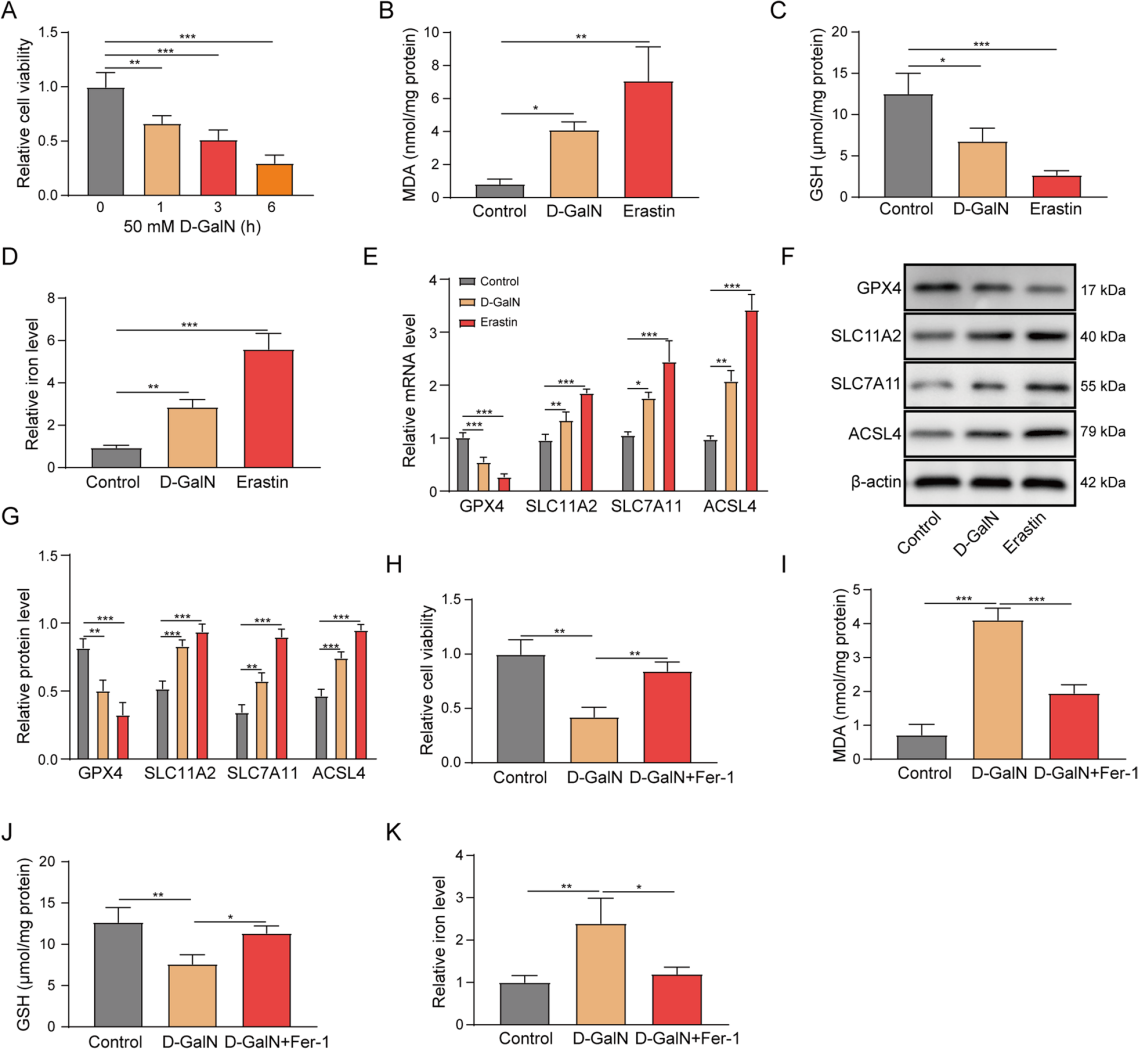
Ferroptosis is activated in D-GalN-induced hepatocyte injury model. **A** Primary hepatocytes were treated with 50 mM D-GalN. Cell viability was monitored by MTT assay. Primary hepatocytes were treated with 50 mM D-GalN or 10 μM erastin for 6 h. **B** MDA, **C** intracellular GSH, and**D** relative iron levels were assessed. **E** The mRNA levels of GPX4, SLC11A2, SLC7A11 and ACSL4 were determined by qRT-PCR. (F and G) The protein levels of GPX4, SLC11A2, SLC7A11 and ACSL4 were determined by western blot. Primary hepatocytes were treated with 50 mM D-GalN or 50 mM D-GalN + 1 μM Fer-1 for 6 h. **H** Cell viability, **I** MDA, **J** GSH and **K** relative iron levels were assessed. *, *P* < 0.05, **, *P* < 0.01, ***, *P* < 0.001

To gain in-depth understanding of the mechanism by which D-GalN induces hepatocyte injury, ferroptosis was evaluated by a series of experiments in primary hepatocytes. As presented in Fig. 1A, D-GalN decreased cell viability of primary hepatocytes time-dependently. We next examined the effects of D-GalN on ferroptosis via assessing the lipid peroxidation product MDA, GSH and iron levels. The well-documented ferroptosis inducer erastin served as a positive control. Similar to erastin, D-GalN significantly increased the levels of MDA and iron, but decreased intracellular GSH level compared to that of control cells (Fig. 1B–D). Moreover, the ferroptosis-related molecules GPX4, solute carrier family 11 member 2 (SLC11A2), SLC7A11 and acyl-CoA synthetase long chain family member 4 (ACSL4) were examined by qRT-PCR and western blot. Interestingly, D-GalN reduced GPX4, but induced SLC11A2, SLC7A11 and ACSL4 expression at both mRNA and protein levels. These effects were similar with erastin (Fig. 1E–G). To further confirm the effects of D-GalN on ferroptosis, the ferroptosis inhibitor Fer-1 were employed in D-GalN-induced hepatocyte injury model. As expected, pre-treatment of Fer-1 markedly abrogated D-GalN-suppressed cell viability and intracellular GSH level, as well as D-GalN-induced MDA and iron levels in primary hepatocytes (Fig. 1H–K). Taken together, these findings suggest that D-GalN triggers ferroptosis in primary hepatocytes.

### HBx potentiates D-GalN-induced ferroptosis.

**Fig. 2:**
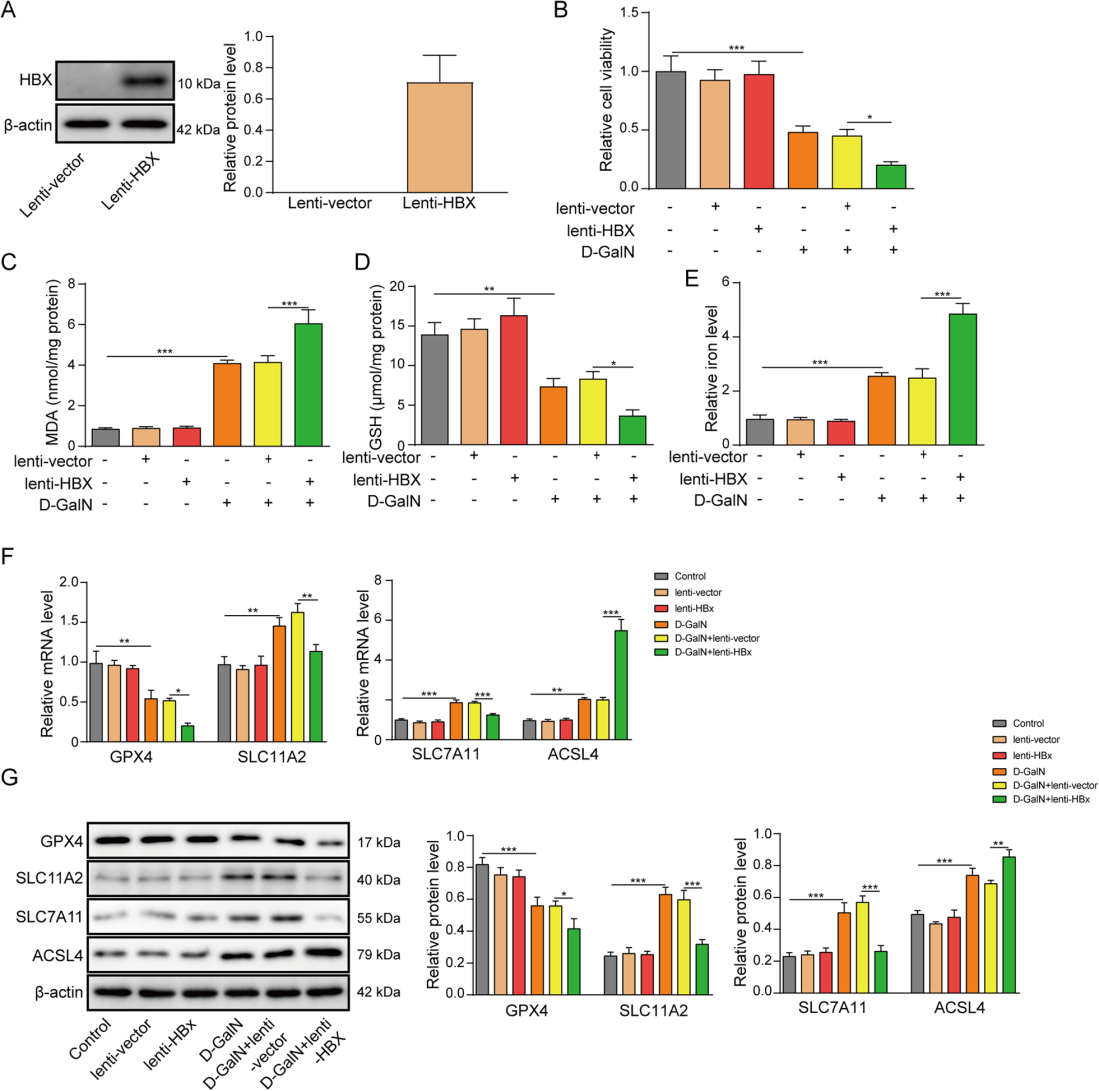
HBx potentiates D-GalN-induced ferroptosis. **A** The protein level of HBx was determined by western blot. Transduced or control cells were subjected to 50 mM D-GalN treatment for 6 h. **B** Cell viability, **C** MDA, **D** GSHand **E** relative iron levels were assessed. **F** The mRNA levels of GPX4, SLC11A2, SLC7A11 and ACSL4 were determined by qRT-PCR. **G** The protein levels of GPX4, SLC11A2, SLC7A11 and ACSL4 were determined by western blot. *, *P* < 0.05, **, *P* < 0.01, ***, *P* < 0.001

In order to explore the biological function of HBx on D-GalN-induced ferroptosis, overexpression experiments were performed in primary hepatocytes. As shown in Fig. 2A, transduction of lenti-HBx significantly upregulated HBx protein level. D-GalN-impaired cell viability was exacerbated by lenti-HBx (Fig. 2B). Overexpression of HBx also remarkably potentiated D-GalN-induced upregulation of MDA and iron, as well as D-GalN-mediated downregulation of GSH (Fig. 2C–E). qRT-PCR and western blot revealed that HBx overexpression potentiated the effects of D-GalN on GPX4 and ACSL4 expression, whereas D-GalN-induced upregulation of SLC11A2 and SLC7A11 were attenuated by lenti-HBx at both mRNA and protein levels (Fig. 2F–G). It is worth noting that transfection of lenti-HBx alone exerted no significant effects on cell viability, MDA, GSH and iron levels, as well as the protein levels of GPX4, SLC11A2, SLC7A11 and ACSL4 (Fig. 2B–G). These data indicate that HBx alone did not trigger ferroptosis in vitro. Collectively, these data indicate that HBx potentiates D-GalN-induced ferroptosis in primary hepatocytes.

### HBx suppresses SLC7A11 expression through H3K27me3 by EZH2.

**Fig. 3:**
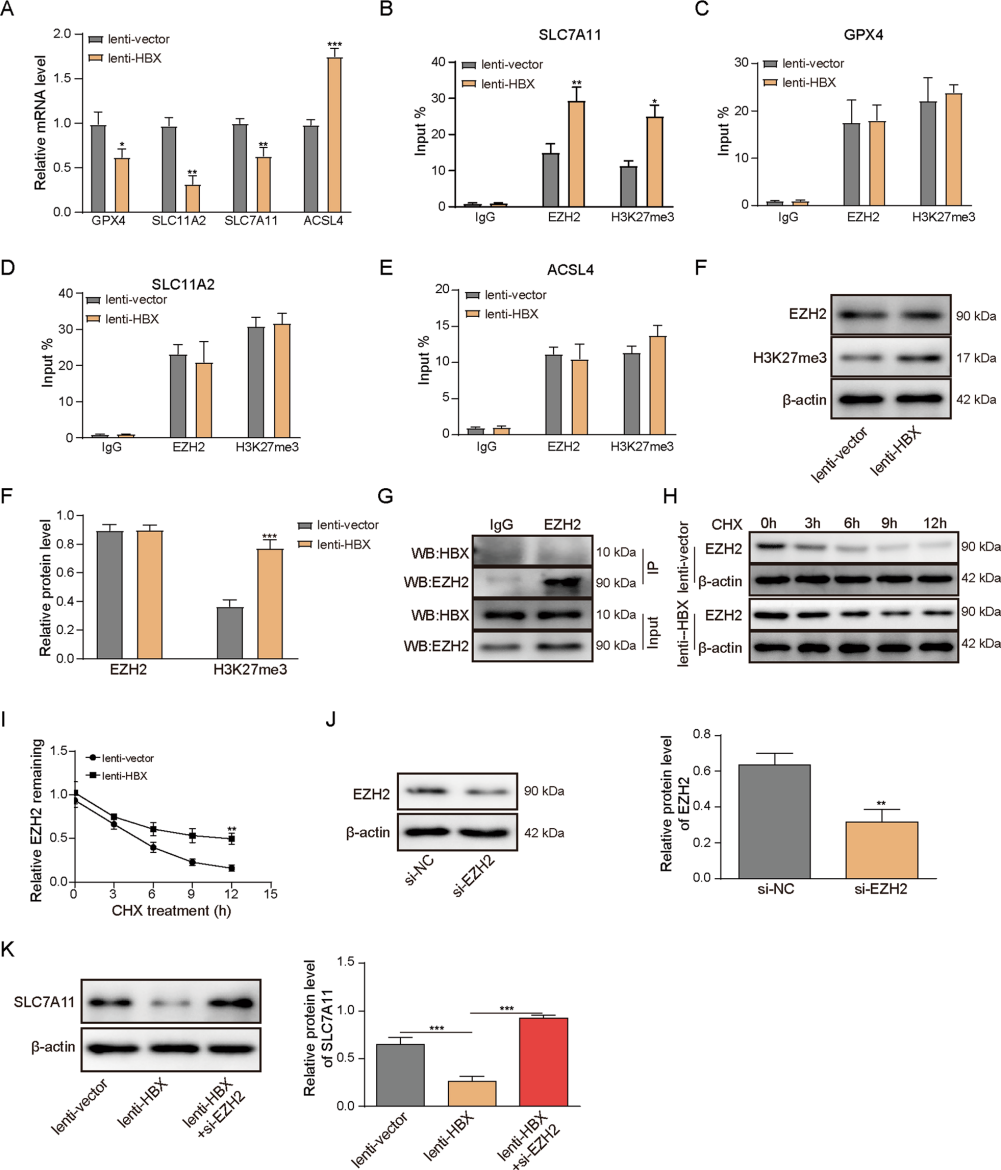
HBx suppresses SLC7A11 expression through H3K27me3 by EZH2. Primary hepatocytes were transduced with lenti-vector or lenti-HBx. **A** The mRNA levels of GPX4, SLC11A2, SLC7A11 and ACSL4 were determined by qRT-PCR. The enrichments of EZH2 and H3K27me3 on promoters of **B** SLC7A11, **C** GPX4, **D** SLC11A2 and **E** ACSL4 were determined by ChIP assay. Normal IgG served as a negative control. **F** The protein levels of EZH2 and H3K27me3 were determined by western blot. **G** The direct interaction between HBx and EZH2 was assessed by co-IP. Normal IgG served as a negative control, and whole cell lysates served as an input control. **H** and **I** Transduced cells were pre-treated with CHX for 1 h. Cells were harvested at 0, 3, 6, 9 and 12 h. The protein level of EZH2 was determined by western blot. **J** and **K** The protein level of EZH2 and SLC7A11 were determined by western blot. *, *P* < 0.05, **, *P* < 0.01, ***, *P* < 0.001

It is well-established that EZH2 functions as a histone methyltransferase to suppress gene expression by trimethylation of H3K27 [25]. To test the aforementioned hypothesis, we investigated the effects of HBx on the ferroptosis-related molecules. In line with the results in D-GalN-induced hepatocyte injury model, overexpression of HBx markedly downregulated the mRNA levels of GPX4, SLC11A2, SLC7A11, but upregulated ACSL4 expression in primary hepatocytes (Fig. 3A). We next examined the enrichments of EZH2 and H3K27me3 in the promoters of these genes by ChIP assay. As presented in Fig. 3B–E, significant increased enrichments of EZH2 and H3K27me3 were found in the promoter region of SLC7A11 upon HBx overexpression, while negligible effects of HBx were observed in GPX4, SLC11A2 or ACSL4 promoters. Thus, the attention was focused on SLC7A11. We sought to delineate the mechanism by which HBx downregulated SLC7A11 via EZH2/H3K27me3. Western blot showed that HBx had no effect on EZH2 expression, whereas H3K27me3 was significantly upregulated in HBx-overexpressing hepatocytes (Fig. 3F). In addition, no direct interaction between HBx and EZH2 was detected by co-IP (Fig. 3G). To further test whether HBx stabilized EZH2 and facilitated H3K27me3 formation, the protein synthesis inhibitor cycloheximide (CHX) was employed to block protein translation. Interestingly, EZH2 was degraded at a significantly slow rate in HBx-overexpressing hepatocytes compared to the control group (Fig. 3H and I). To further support of this mechanism, EZH2 knockdown experiments were performed. As shown in Fig. 3J, transfection of si-EZH2 successfully downregulated EZH2 protein level. Overexpression of HBx decreased SLC7A11 expression in the presence of D-GalN, whereas silencing of EZH2 resulted in a marked rebound of SLC7A11 in comparison with corresponding control (Fig. 3K). These findings indicate that HBx stabilizes EZH2 and facilitates trimethylation of H3K27, thereby suppressing SLC7A11 in primary hepatocytes.

### EZH2 inhibition or SLC7A11 overexpression reverses the effects of HBx on D-GalN-induced ferroptosis.

**Fig. 4:**
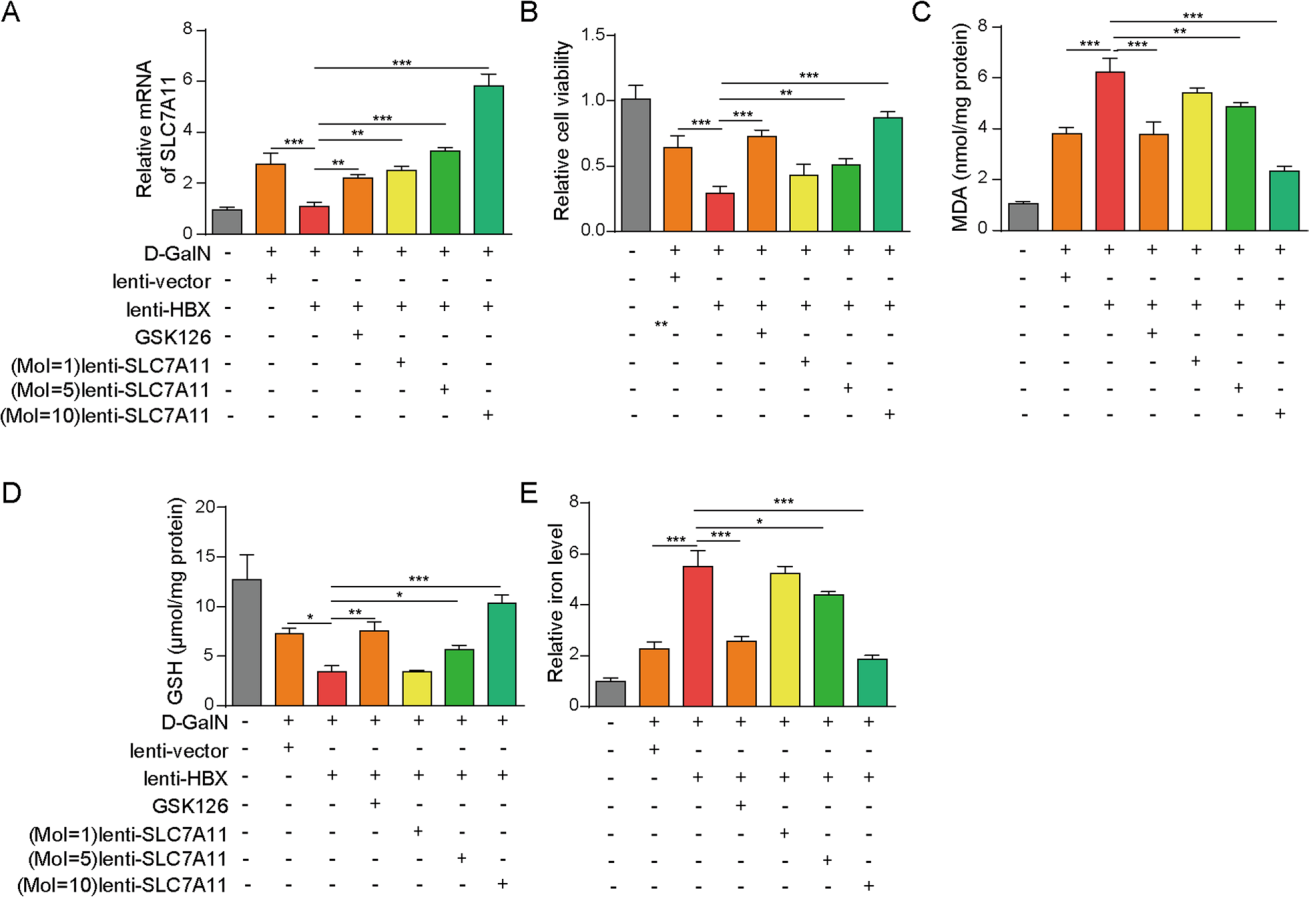
EZH2 inhibition or SLC7A11 overexpression reverses the effects of HBx on D-GalN-induced ferroptosis. Transduced cells were treated with 50 mM D-GalN in the absence or presence of GSK126 (5 μM)/SLC7A11 overexpression vector (MOI = 1, 5, 10) for 48 h. **A** The mRNA level of SLC7A11 was determined by qRT-PCR. **B** Cell viability, **C** MDA, **D** GSHand **E** iron levels were assessed *, *P* < 0.05, **, *P* < 0.01, ***, *P* < 0.001

In order to further validate the role of HBx-mediated SLC7A11 suppression in D-GalN-induced ferroptosis, gain- and loss-of function experiments were performed in D-GalN-induced hepatocyte injury model. In accordance with previous data, overexpression of HBx attenuated D-GalN-mediated upregulation of SLC7A11, whereas GSK126 led to rebound of SLC7A11 expression compared to D-GalN + lenti-HBx group (Fig. 4A). As expected, lenti-SLC7A11 transduction successfully rescued lenti-HBx-downregulated SLC7A11 mRNA level dose-dependently (Fig. 4A). We further investigated the effects of GSK126 or lenti-SLC7A11 on the ferroptosis-related parameters. Consistently, HBx overexpression potentiated D-GalN-mediated impairment of cell viability, elevated levels of MDA and iron, and D-GalN-induced downregulation of GSH level. GSK126 or SLC7A11 overexpression markedly reversed these effects of HBx on cell viability, MDA, GSH and iron levels (Fig. 4B–E). It is worth noting that lenti-SLC7A11 exhibited these effects in a dose-dependent manner, and the effects of lenti-SLC7A11 (MOI = 10) was more prominent than the lower doses (Fig. 4B–E). Taken together, these data suggest that EZH2 inhibition or SLC7A11 overexpression reverses the effects of HBx on D-GalN-induced ferroptosis.

### Fer-1 protects against LPS/D-GalN-induced ALF and ferroptosis in mice.

**Fig. 5:**
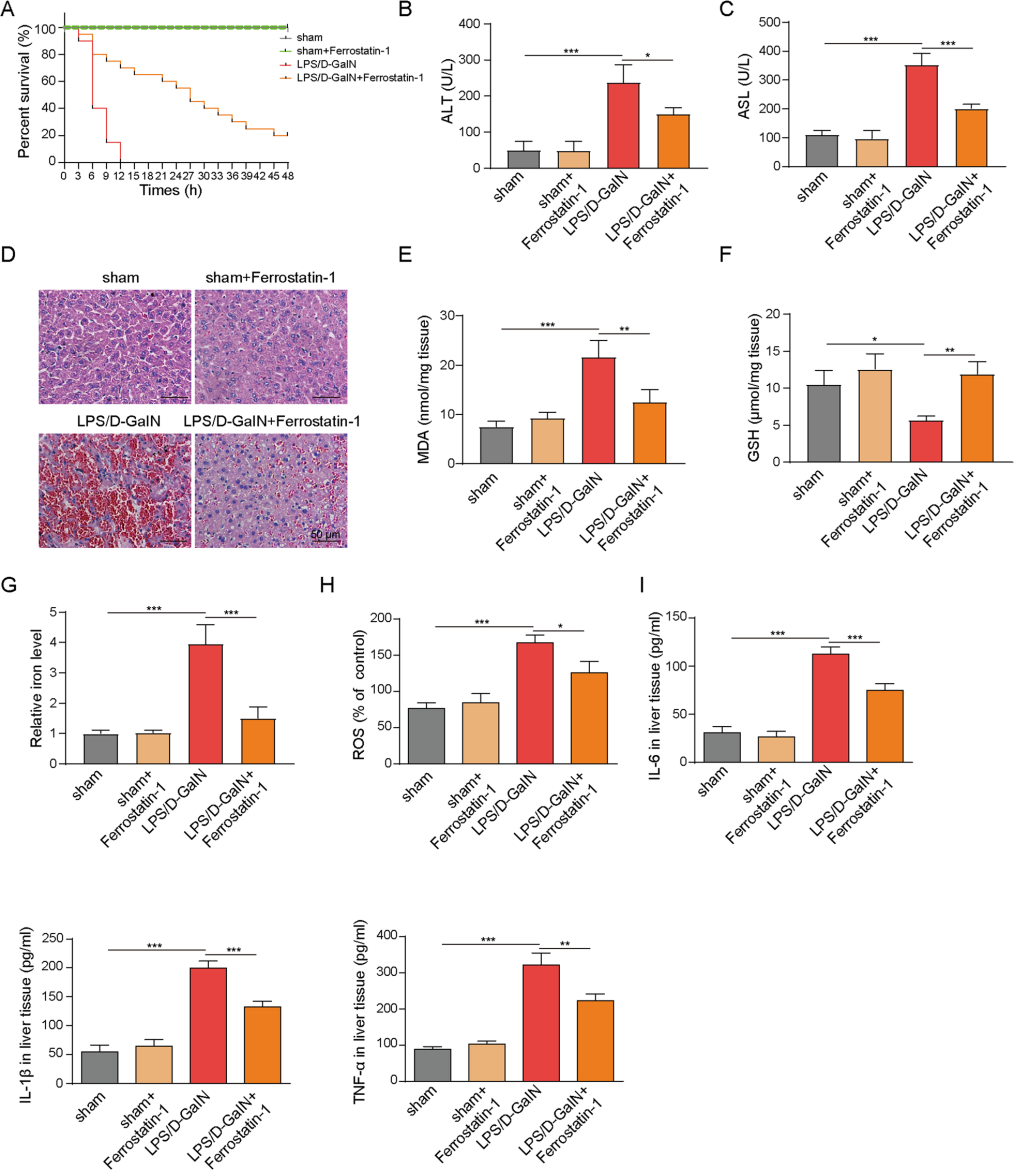
Fer-1 protects against LPS/D-GalN-induced ALF in mice. **A** The survival curve of sham, sham + Fer-1, LPS/D-GalN and LPS/D-GalN + Fer-1 mice. The serum levels of **B** ALT and **C** AST were assessed. **D** The histopathological changes were determined by H&E staining. The levels of **E** MDA, **F** GSH, **G** iron, **H** ROS and **I** TNFα, IL-6, IL-1β levels were assessed. *, *P* < 0.05, **, *P* < 0.01, ***, *P* < 0.001

To further validate the important role of ferroptosis in vivo, the LPS/D-GalN-induced ALF mice model was generated. Fer-1 was employed to inhibit ferroptosis during ALF. All mice in sham and sham + Fer-1 groups survived within 48 h. Notably, Fer-1 dramatically ameliorated ALF-induced death in which the survival rate of LPS/D-GalN + Fer-1 was ~ 20% within 48 h. By contrast, all mice in LPS/D-GalN group died within 12 h (Fig. 5A). Compared to sham and sham + Fer-1 groups, the ALT and AST activities were remarkably increased in LPS/D-GalN mice, confirming the successful establishment of ALF model. Pre-treatment of Fer-1 significantly abrogated LPS/D-GalN-induced ALT and AST activities (Fig. 5B, C). H&E staining revealed that regular hepatic sinusoidal structure and clear hepatic lobules were observed in liver tissues of sham and sham + Fer-1 mice, whereas cell edema, inflammatory cell infiltration and sever intrahepatic hemorrhage were found in LPS/D-GalN mice. Consistently, LPS/D-GalN-induced hepatotoxicity was partially rescued by Fer-1 (Fig. 5D), suggesting that ferroptosis plays a crucial role during ALF. Moreover, we next examined the levels of MDA, GSH and iron in liver tissue. In accordance with the in vitro data, Fer-1 reversed LPS/D-GalN-mediated MDA and iron elevation, as well as LPS/D-GalN-decreased GSH in vivo (Fig. 5E–G). Furthermore, ROS detection and ELISA assays revealed that Fer-1 remarkably attenuated LPS/D-GalN-increased ROS, TNF-α, IL-6 and IL-1β levels in liver tissues, while Fer-1 alone had no significant effect on the levels of ROS or these inflammatory cytokines (Fig. 5H–I). Hence, these data implicate ferroptosis as an important contributor in ALF.

### HBx exacerbates LPS/D-GalN-induced ALF and ferroptosis in vivo.

**Fig. 6:**
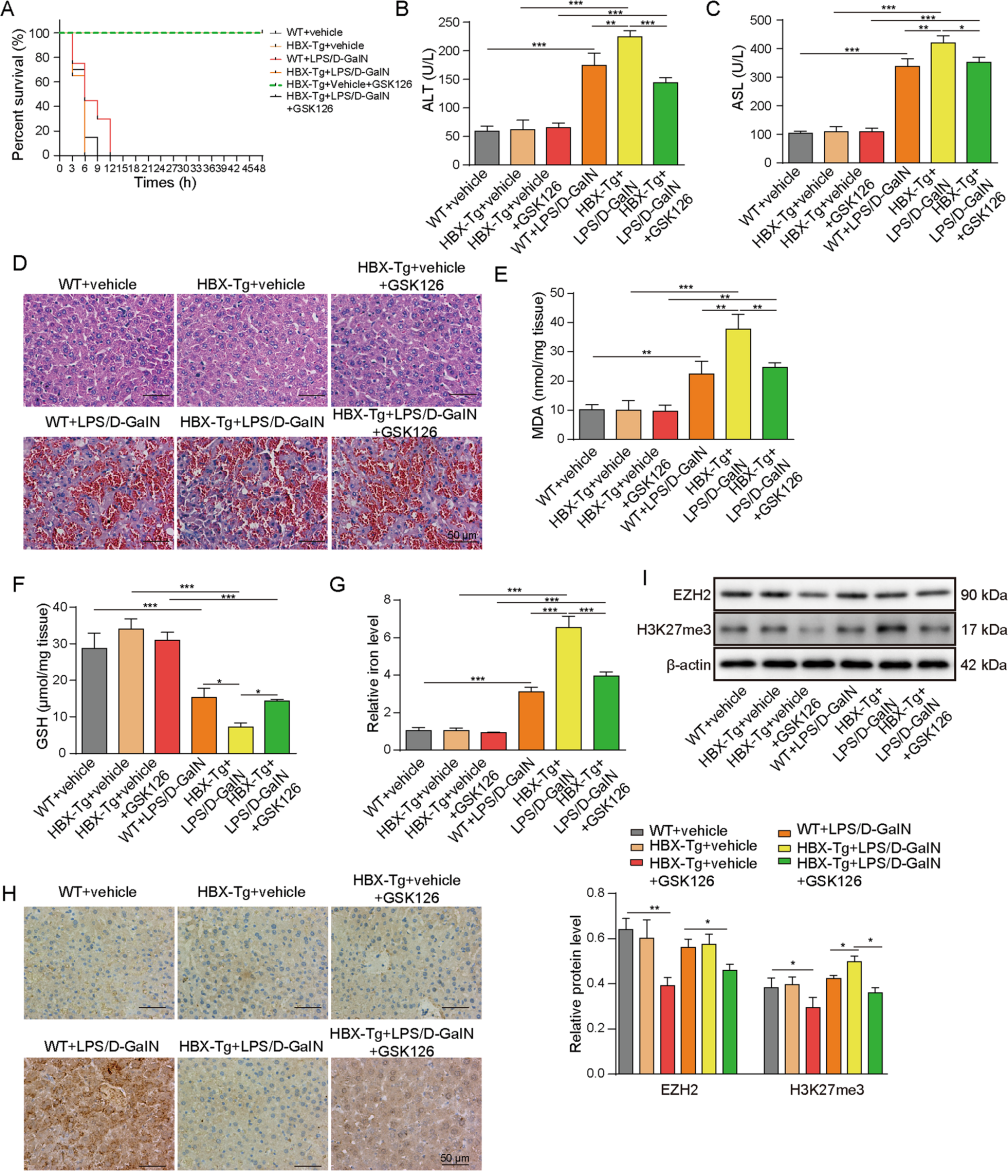
HBx exacerbates LPS/D-GalN-induced ALF and ferroptosis in vivo. **A** The survival curve of WT + vehicle, HBx-Tg + vehicle, WT + LPS/D-GalN and HBx-Tg + LPS/D-GalN mice. The serum levels of **B** ALT and **C** AST were assessed. **D** The histopathological changes were determined by H&E staining. The levels of **E** MDA, **F** GSHand, **G** iron were assessed. **H** The immunoreactivity of SLC7A11 was determined by IHC analysis. **I** The protein levels of EZH2 and H3K27me3 in liver tissues were determined by western blot. *, *P* < 0.05, **, *P* < 0.01, ***, *P* < 0.001

We next validated whether HBx played a critical role in ALF using HBx transgenic (HBx-Tg) mice. The wild-type (WT) and HBx-Tg mice were subjected to vehicle or LPS/D-GalN treatment, as well as GSK126 administration. All mice in WT + vehicle, HBx-Tg + vehicle and HBx-vehicle + GSK126 groups survived within 48 h, whereas all mice in WT + LPS/D-GalN, HBx-Tg + LPS/D-GalN and HBx + LPS/D-GalN + GSK126 groups died within 12 h. There was no significant difference in mortality among WT + vehicle, HBx-Tg + vehicle and HBx-Tg + vehicle + GSK126 groups (Fig. 6A). Intriguingly, survival of HBx-Tg + LPS/D-GalN mice declined faster than WT + LPS/D-GalN and HBx-Tg + LPS/D-GalN + GSK126 mice (Fig. 6A). To assess the liver injury, the serum ALT and AST activities were measured. As shown in Fig. 6B, C, LPS/D-GalN-induced ALT and AST activities were further potentiated in HBx-Tg + LPS/D-GalN mice, whereas GSK126 partially attenuated this effect. Consistent with previous study, liver tissues from HBx-Tg mice exhibited moderate chronic hepatitis, hepatic necrosis and intracytoplasmic fat vacuoles [26]. Liver tissues from WT + LPS/D-GalN mice showed similar histological changes with LPS/D-GalN mice. Increased inflammation, severer hepatocytes swelling and intrahepatic hemorrhage were observed in HBx-Tg + LPS/D-GalN mice (Fig. 6D), indicating that HBx exacerbates LPS/D-GalN-induced ALF. Similarly, the exacerbated histological impairment was ameliorated by GSK126 (Fig. 6D). Moreover, LPS/D-GalN-induced changes of MDA, GSH and iron levels in liver tissue were also potentiated in HBx-Tg + LPS/D-GalN mice, while GSK126 exhibited protective effects on MDA, GSH and iron levels (Fig. 6E–G), which was consistent with the in vitro findings. Interestingly, the immunoreactivity of SLC7A11 was significantly elevated in the liver tissues of WT + LPS/D-GalN mice in comparison with that of WT + vehicle mice. By contrast, HBx-Tg + LPS/D-GalN mice exhibited no remarkable change in SLC7A11 level compared to that of HBx-Tg + vehicle mice (Fig. 6H). GSK126 administration led to a rebound of SLC7A11 in liver tissues, compared with HBx-Tg + LPS/D-GalN mice (Fig. 6H). Additionally, EZH2 and H3K27me3 levels in the liver tissues were also examined. As shown in Fig. 6I, no significant difference in EZH2 levels were observed between WT + vehicle and WT + LPS/D-GalN mice, as well as between HBx-Tg + vehicle and HBx-Tg + LPS/D-GalN mice. H3K27me3 was slightly upregulated in WT + LPS/D-GalN mice, compared with WT + vehicle mice. The upregulation of H3K27me3 is more prominent in HBx-Tg + LPS/D-GalN mice, and GSK126 partially abolished LPS/D-GalN-increased H3K27me3 expression in both WT and HBx-Tg mice. Taken together, these findings suggest that HBx exacerbates LPS/D-GalN-induced ALF and ferroptosis in vivo, possibly via suppressing SLC7A11.

## Discussion

In the present study, we illustrated that ferroptosis played a critical role in D-GalN-induced hepatocyte injury and LPS/D-GalN-induced ALF mice model. Mechanistic study also revealed that HBx facilitated ferroptosis via EZH2/H3K27me3-mediated SLC7A11 suppression, thereby exacerbating ALF.

Ferroptosis was first identified as a novel form of cell death in cancer [4]. In recent years, emerging evidence suggest that ferroptosis is involved in the pathogenesis of different types of liver diseases, including HCC, viral hepatitis, non-alcoholic steatohepatitis (NASH) and alcoholic liver disease (ALD), as well as ALF [6–8, 27–31]. Ferroptosis is characterized by iron-dependent accumulation of lipid ROS. Therefore, researches have become interested in the imbalance of iron metabolism and ROS-induced lipid peroxidation in these diseases. The HMGB1 inhibitor glycyrrhizin alleviates ferroptosis in ALF through inhibiting oxidative stress [6]. A more recent study has demonstrated that ferroptosis is responsible for hepatocyte cell death in acetaminophen-induced ALF [8]. However, the mechanisms of ferroptotic hepatocyte cell death remain elusive. In accordance with previous studies, our findings suggested that D-GalN-induced hepatotoxicity was accompanied with iron accumulation and lipid peroxidation, whereas these effects of D-GalN was abrogated by Fer-1. These findings were also validated in a murine ALF model induced by LPS/D-GalN. Moreover, LPS/D-GalN-caused lethality was partially rescued by Fer-1 in vivo. These data indicate that ferroptosis might be a promising therapeutic target for ALF treatment.

HBx, a multifunction protein, is involved in diverse biological processes, including transcriptional regulation, apoptosis, cell growth arrest, cell cycle progression and protein degradation, as well as induction of oxidative stress [16, 17, 32, 33]. In human hepatoma cell line HepG2 cells, HBx induces lipid peroxidation through suppressing selenoprotein P, thereby increasing TNFα expression [16]. In addition, HBx is also implicated in mitochondrial ROS and lipid peroxidation production. It is noteworthy that increased intrahepatic lipid peroxidation was also observed in HBx-Tg mice [17]. Recent studies support a critical role of lipid peroxidation and mitochondrial ROS in ferroptosis [5, 34], suggesting that HBx might contribute to pathogenesis of liver diseases by regulating ferroptosis. In this study, our findings has first established HBx as an important regulator of ferroptosis in ALF. In D-GalN-induced hepatocyte injury model, we found that HBx overexpression potentiated D-GalN-induced lipid peroxidation, GSH depletion and iron accumulation. These findings were also observed in LPS/D-GalN-treated HBx-Tg mice, confirming the crucial role of HBx-regulated ferroptosis in ALF.

SLC7A11 is an amino acid antiporter which responsible for extracellular cysteine uptake in exchange for glutamate. Impaired cysteine uptake inhibited GSH biosynthesis, further resulting in loss of GPX4 activity [4, 5]. It is well-accepted that lack of GPX4 triggers ferroptosis via increased accumulation of lipid peroxidation [35]. In the present study, our data suggested that both D-GalN and erastin upregulated SLC11A2 and SLC7A11 in primary hepatocytes. These findings were in agreement with a previous report which has demonstrated that erastin induces SLC7A11 expression as an adaptive response [36], indicating that D-GalN-induced upregulation of SLC11A2 and SLC7A11 might also be a compensatory effect on system Xc^−^ inhibition. Moreover, the tumor suppressors p53 and BRCA1-associated protein 1 (BAP1) has been identified as upstream regulators of SLC7A11 during ferroptosis [37, 38]. Our study implicates HBx as an important regulator of SLC7A11 in ALF. Mechanistic studies revealed that HBx suppresses SLC7A11 expression through EZH2/H3K27me3. HBx promoted protein stability of EZH2, and the most prominent effects of HBx were observed at 6 and 9 h post-CHX treatment, while these effects become negligible over time. This is the possible reason why no significant effect of HBx was observed on EZH2 protein level 24 h post-transduction. In addition, our data did not favor a direct interaction between HBx and EZH2. Previous study has demonstrated that HBx upregulates lncRNA UCA1 which is physically interacted with EZH2, thus suppressing p27Kip via trimethylation of H3K27 [20]. We speculate that lncRNA might act as a mediator to facilitate the recruitment of EZH2 and H3K27me3 in the promoter region of SLC7A11. Identification of the mediator merits further investigation. Growing evidence has illustrated that HBx-induced aberrant epigenetic regulation in DNA methylation, histone modifications and miRNA alterations play critical roles in HCC [19]. Besides the well-characterized roles in HCC, our findings in ALF introduces a broader view of HBx-induced aberrant epigenetic regulation. However, HBx is known to induce various types of epigenetic modifications, such as H3K4 methylation and DNA hypermethylation [19, 39]. Different HBx-induced epigenetic modifications which contributes to ferroptosis in ALF merit further investigation.

## Conclusions

In conclusion, HBx facilitates ferroptosis in ALF via EZH2/H3K27me3-mediated SLC7A11 suppression.

## Data Availability

The raw data supporting the conclusions of this manuscript will be made available by the authors, without undue reservation, to any qualified researcher.

## Abbreviations

ACSL4: Acyl-CoA synthetase long chain family member 4
ALD: Alcoholic liver disease;
ALF: Acute liver failure
ALT: Alanine aminotransferase
AST: Aspartate aminotransferase
ChIP: Chromatin immunoprecipitation
CHX: Cycloheximide
co-IP: Co-immunoprecipitation
D-GalN: D-galactosamine
EZH2: Enhancer of zeste homolog 2
Fer-1: Ferrostatin-1
GPX4: Glutathione peroxidase 4
GSH: Glutathione
H3K27me3: Histone H3 lysine 27 tri-methylation
HBV: Hepatitis B virus
HBx: Hepatitis B virus X protein
HBx-Tg: HBx transgenic
HCC: Hepatocellular carcinoma
H&E: Hematoxylin and eosin
IHC: Immunohistochemistry
LPS: Lipopolysaccharide
MDA: Malondialdehyde
NASH: Non-alcoholic steatohepatitis
ROS: Reactive oxygen species
SLC7A11: Solute carrier family 7 membrane 11
SLC11A2: Solute carrier family 11 member 2
